# Analysis of Laminated Composite Porous Plate under Sinusoidal Load with Various Boundary Conditions

**DOI:** 10.3390/ma17102308

**Published:** 2024-05-13

**Authors:** Raushan Kumar, Ajay Kumar, Wojciech Andrzejuk, Małgorzata Szafraniec, Danuta Barnat-Hunek

**Affiliations:** 1 Department of Civil Engineering, Gaya College of Engineering, Gaya 823003, India; 2 Department of Civil Engineering, National Institute of Technology Delhi, Delhi 110036, India; sajaydce@gmail.com; 3 Faculty of Technical Sciences, John Paul II University in Biala Podlaska, Sidorska 95/97, 21-500 Biała Podlaska, Poland; w.andrzejuk@dyd.akademiabialska.pl; 4 Faculty of Civil Engineering and Architecture, Lublin University of Technology, Nadbystrzycka 40, 20-618 Lublin, Poland; m.szafraniec@pollub.pl

**Keywords:** bending analysis, porous plate, finite element, laminated plate, numerical example

## Abstract

Bending analysis was carried out for a laminated composite porous plate due to sinusoidal loading with various boundary conditions using improved third-order theory. Zero transverse shear stress provided a free surface at the top and bottom of the plate. Also, the authors developed a finite element formulation based on improved third-order shear deformation theory. To circumvent the C1 continuity requirement associated with improved third-order shear deformation theory, a C0 FE formulation was developed by replacing the out-of-plane derivatives with independent field variables. An in-house FORTRAN code was developed for the bending analysis of the laminated porous plate considering a 2D finite element model. The complete thickness of the plate was covered with different porosity patterns. The impacts of various modulus ratios, boundary conditions, thickness ratios, fiber orientation angles, and material parameters were examined for laminated porous plates. There was an 18.8% reduction in deflection in the case of the square plate as compared to rectangular plates, with a porosity value of 0.1, a thickness ratio of 10, and an orientation angle of 0°/90°/0°. According to the current research, adding porosities causes a relatively greater change in deflection rather than stress, thereby aiding in the development of a lightweight structure.

## 1. Introduction

Multiple types of materials are combined to create laminated composites. Different materials have been extensively utilized to create strengthened composites for the automotive, marine, and aeronautical sectors in recent years. The outstanding stiffness-to-weight ratio of laminated composites make them a popular choice for applications in mechanical and civil engineering. In solid mechanics, bending is an extremely important phenomenon. For the construction of beams, cars, spacecraft, etc., bending analysis is applied. Many investigators have utilized different boundary conditions, thickness ratios, modulus ratios, orientation angles, material characteristics, etc., to perform bending analyses of layered plates [1]. In-depth discussions on the topic can be found in the available research.

Kant and Swaminathan [2] presented a numerical solution for the flexural behavior of multilayered sheets and sandwiched plates, considering the transverse shear effect of the higher-order deformation approach. In order to create magnetostrictive patches that function as both sensors and accuators, Ghosh and Gopalakrishnan [3] studied a unique analytical method for laminated composite. Liu et al. [4] used an isoparametric method to conduct flexural and dynamic analyses of the multilayer plate, utilizing a variety of mess-free techniques. Vidal and Polit [5] conducted a vibration and flexural study of a multilayered beam using a three-noded element and a sinus technique. Flexural analyses of multilayered plates with different edge conditions were examined by Naserian and Tahani [6]. Due to aerodynamic load, Mahato and Maiti [7] conducted research on the aeroelastic behavior of smart layer composites. Pandit et al. [8] used an enhanced higher-order zigzag method to analyze the flexural and dynamic behavior of the switched plate.

Using a novel higher-order deformation approach, Mantari et al. [9] performed flexural and vibration analysis of shells, layered composites, and switched plates. Moita et al. [10] investigated the normal frequency of the laminated sandwiched sheet by considering the core’s viscoelastic properties and the inner portion’s elastic properties. Zaman et al. [11] used a mixture of chemical transformation and sonication to produce two kinds of epoxy/GP nanocomposites with varying interface strengths. Using an analytical technique, Narayana et al. [12] focused on how laminated composite sheets with rectangular perforations and different in-plane stresses buckled. A flexural study of several laminated composite sheets that had different boundary circumstances was carried out by Rango et al. [13] using the first-order deformation approach. Reddy et al. [14] utilized the approach of finite elements to study the static analysis of a multilayered plate while taking transverse shear effects into account.

Vanam et al. [15] used an analytical approach to study the flexural responses of multilayer composite plates with different edge circumstances. A cross-ply multilayered plate’s bending analysis was examined by Ghugal and Kulkarni [16] because of nonlinearly changing temperatures and loading effects. Ramos et al. [17] examined how inadequate contact circumstances can be caused by natural factors, manmade interface designs, or chemical interactions between the fiber and matrix substance. Ferreira et al. [18] analyzed the dynamic responses of thin and thick cross-ply using a unified technique. Grover et al. [19] created a secant-based tangential deformation analytical model for the bending analysis of the sandwiched structure. For the flexural analysis of switched structures, Sahoo and Singh [20] created a novel trigonometric zigzag method that takes nonlinear strain distribution over thickness into account.

Using a variety of deformation theories, Sayyad et al. [21] examined the thermoelastic bending behavior of laminated plates under sinusoidal linear changing loads. Hirwani et al. [22] conducted a numerical approach to the bending behavior of delaminated composite plates, and experimental validation research was conducted. A unique higher-order deformation technique was created by Sadiq and Abdul-Ameer [1] to study the flexural behavior of multilayered composite structures. Gopinath and Batra [23] looked at combining techniques to create fiber-reinforced elasto-plastic composite materials. Their techniques included the use of cellular approaches, Fourier series, and transformation techniques. The impact of chemical groups on graphene accumulation in nanocomposites was examined by Li et al. [24]. To explain the fluctuating and balanced characteristics of laminated composites with spatially variable micro- and macro-mechanical component features, Naskar et al. [25] proposed an unpredictable framework.

Demirhan and Taskin [26] used four variable plate hypotheses with simply supported edge circumstances to examine the flexural behavior of functionally graded plates. Kumar et al. [27] examined the influence of obliqueness in the strike angle and angular distortion in the sheet shape on the low-velocity collision behaviors of sandwich plates with laminate face layers. Adhikari and Singh [28] analyzed the vibration responses of the layered sheet with varying edge loads. An analytical method was developed by Chanda and Sahoo [29] for the flexural study of sandwiched plates and multilayered sheets. According to Fantuzzi et al. [30], carbon nanotubes can strengthen the polymeric matrix as well as enhance the mechanical characteristics of the resultant composite. A flexural study was conducted by Patel and Sharma [31] on a laminated composite plate with a polygonal cutout. The results rely on the loading distribution, number of layers, fiber orientation, hole shape, and corner radius. Hoang [32] performed the interpolation technique with varying meshing divisions for the laminated plate. Zenkour and El-Shahrany [33] investigated the dynamic responses of a sandwiched plate resting on an elastic base. Belardi et al. examined the mechanical properties of a laminated sector plate with rectilinear orthotropy, specifically focusing on deflection and stresses inside the laminated composite plate [34]. Algül and Oktem [35] examined the flexural analysis of symmetric and antisymmetric laminated plates using a double Fourier series. Chanda et al. [36] studied the static and dynamic behavior of a multilayered smart piezoelectric plate resting on an elastic base. Assie et al. [37] developed a mathematical model for the static analysis of a bi-directional pervious plate resting on elastic foundation, with the help of an efficient mathematical approach including a differential integral quadrature technique. Bab and Kutlu [38] applied a C^0^ efficient model to analyze the stresses of laminated composite plate, with the help of higher-order deformation theory. Tran et al. [39] developed a basic equation based on higher-order shear deformation theory for the flexural analysis of pervious plates. Tru et al. [40] conducted static analysis of functionally graded pervious plates with the help of naval shear deformation theory. They considered uniform and non-uniform porosity distribution over the entire thickness of the plate.

In this study, porosity influence is investigated through bending analysis of the laminated plate’s thickness. The influence is also noted for different orientation angles, material characteristics, boundary circumstances, and thickness ratios, among other elements. The first-, second-, and third-order shear deformation hypotheses are only a few of the techniques used in the literature mentioned above for composite plate bending analysis. Classical plate theory is also used for analysis. Limited research has been done on the bending analysis of laminated composite porous plates. For the bending analysis of multilayered plates, no research has used an improved third-order shear deformation concept with various boundary conditions and porosity effects due to sinusoidal loading. Employing in-house FORTRAN code, we conducted bending analysis for a number of 2D finite element technique instances. Improved third-order deformation theory is used to compute all outcomes.

The current study’s objectives are to develop an accurate and efficient 2D finite element approach for evaluating the bending behavior of multilayer composite pervious plates. This study also examines the bending behavior modelling of the layered porous composite plate for various boundary conditions, orientation angles, thickness ratios, and modulus ratios, applying the improved third-order shear deformation hypothesis based on the identified gap in the existing research. After reviewing the literature, the following study topics were identified as significant. The literature review makes it abundantly evident that very little study has been conducted on the examination of layered composite porous plates. No study has been conducted on the bending analysis of a porous layered plate that has a varied boundary circumstance, orientation angle, modulus ratio, and thickness ratio using an improved third-order concept. The bending study of layered composite porous plates with various boundary conditions due to sinusoidal load has been the subject of very few investigations. The literature study highlights the need for a precise and effective model in order to understand how porosity affects the behavior of laminated composite plates.

The novelty of the present research is its addressing of the shortcomings of previous plate theories. We consider transverse shear stress continuity at each layer interface and zero transverse shear stress at the top and bottom of the plate. Also, the authors develop a finite element formulation based on improved third-order shear deformation theory. In-house FORTRAN code was developed to study the bending analysis of laminated composite porous plates.

## 2. Materials and Methods

### 2.1. Relation between Stress and Strain

**Figure 1:**
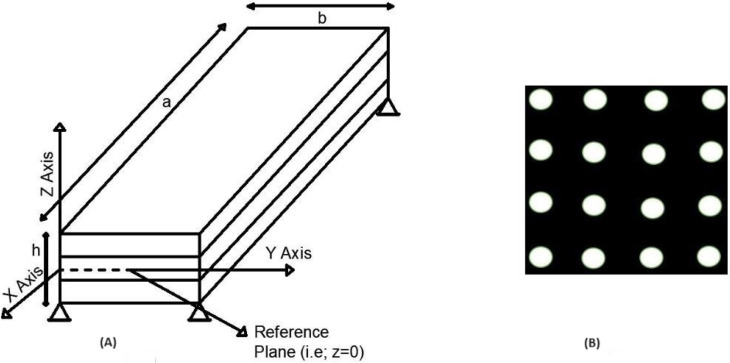
(**A**) Triple-layer simply supported plate and (**B**) dispersion of porosity.

As seen in Figure 1A, a rectangular layered composite plate is presented, with thickness in the z axis and length in the x and y directions, designated L_x_ and L_y_, correspondingly. The figure presents a simply supported plate with sinusoidal and uniformly distributed load. The plate is separated into three sections with identical thickness and uniformity around the middle plane, each of which has three lamina that are oriented at various angles. The whole layer thickness of the plate induces a porosity dispersion, which is represented by the porosity dispersion model in Figure 1B. The material characteristics of the lamina (E) vary in accordance with this model, with the Young’s modulus, Poisson’s ratio, and other parameters being expressed as E(p) = E(1 − p). Numerous instances involving the bending analysis of multilayered pervious plates under sinusoidal stress are addressed in the present work. Convergence and validation analyses are conducted in this work to assess the validity and application of the findings. Furthermore, the full thickness of the plate is filled with porosities, such as 0.1, 0.2, and 0.3. The link between stress and strain is examined as follows.

(1) $$\left\{\begin{array}{l} \sigma_{x}\\ \sigma_{y}\\ \tau_{xy}\\ \tau_{xz}\\ \tau_{yz} \end{array}\right\}=\left[\begin{array}{ccccc} {\bar{Q}}_{11}(p) & {\bar{Q}}_{12}(p) & {\bar{Q}}_{16}(p) & 0 & 0\\ {\bar{Q}}_{12}(p) & {\bar{Q}}_{22}(p) & {\bar{Q}}_{26}(p) & 0 & 0\\ {\bar{Q}}_{16}(p) & {\bar{Q}}_{26}(p) & {\bar{Q}}_{66}(p) & 0 & 0\\ 0 & 0 & 0 & {\bar{Q}}_{55}(p) & {\bar{Q}}_{45}(p)\\ 0 & 0 & 0 & {\bar{Q}}_{45}(p) & {\bar{Q}}_{44}(p) \end{array}\right]\left\{\begin{array}{l} \varepsilon_{x}\\ \varepsilon_{y}\\ \gamma_{xy}\\ \gamma_{xz}\\ \gamma_{yz} \end{array}\right\}\mathrm{or}\;\left\{\bar{\sigma }\right\}=\left[Q^{-i}\right]\left\{\bar{\varepsilon }\right\}$$

Here, the material characteristics ($E_{1},$ $E_{2}$, $\nu_{12}$, $G_{13}$, $G_{23}{,\;G}_{12}$) and stiffness matrix $\left[Q^{-i}\right]$ may be constructed using the lamina fiber orientation (θ) [41]. In Equation (1), (σ*_x_*, σ*_y_*, τ*_xy_*, τ*_xz_*, τ*_yz_*) are the stresses and (ε*_x_*, ε*_y_*, γ*_xy_*, γ*_xz_*, γ*_yz_*) are the strains with respect to the lamina axis. Q_ij_ represents the transform elastic constants or the stiffness matrix.

(2) $${\bar{Q}}_{11}(p)=Q_{11}(p)\cos^{4}\theta +2(Q_{12}(p)+2Q_{66}(p))\sin^{2}\theta \cos^{2}\theta +Q_{22}(p)\sin^{4}\theta \;{\bar{Q}}_{12}(p)=(Q_{11}(p)+Q_{22}(p)-4Q_{66}(p))\sin^{2}\theta \cos^{2}\theta +Q_{12}(p)(\cos^{4}\theta +\sin^{4}\theta )\;{\bar{Q}}_{22}(p)=(Q_{22}(p)\cos^{4}\theta +2(Q_{12}(p)+2Q_{66}(p))\sin^{2}\theta \cos^{2}\theta +Q_{11}(p)\sin^{4}\theta )\;{\bar{Q}}_{16}(p)=(Q_{11}(p)-Q_{12}(p)-2Q_{66}(p))\sin\theta \cos^{3}\theta +(Q_{12}(p)-Q_{22}(p)+2Q_{66}(p))\sin^{3}\theta \cos\theta \;{\bar{Q}}_{26}(p)=(Q_{11}(p)-Q_{12}(p)-2Q_{66}(p))\sin^{3}\theta \cos\theta +(Q_{12}(p)-Q_{22}(p)+2Q_{66}(p))\sin\theta \cos^{3}\theta \;{\bar{Q}}_{66}(p)=(Q_{11}(p)+Q_{22}(p)-2Q_{12}(p)-2Q_{66}(p))\sin^{2}\theta \cos^{2}\theta +{\bar{Q}}_{66}(p)(\cos^{4}\theta +\sin^{4}\theta )\;{\bar{Q}}_{44}(p)=G_{13}(p)\cos^{2}\theta +G_{23}(p)\sin^{2}\theta ,\;{\bar{Q}}_{45}(p)=(G_{13}(p)-G_{23}(p))\sin\theta \cos\theta \;{\bar{Q}}_{55}(p)=G_{23}(p)\cos^{2}\theta +G_{13}(p)\sin^{2}\theta \;Q_{11}(p)=\frac{E_{1}(p)}{1-\nu_{12}(p)\nu_{21}(p)},Q_{12}=\frac{\nu_{12}(p)E_{2}(p)}{1-\nu_{12}(p)\nu_{21}(p)},Q_{22}=\frac{E_{1}(p)}{1-\nu_{12}(p)\nu_{21}(p)},Q_{66}=G_{12}(p).$$

### 2.2. Relation of Displacement and Material Properties

**Figure 2:**
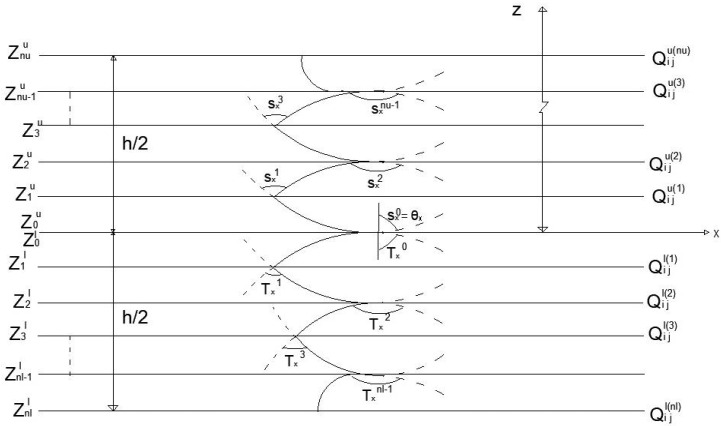
Variations in the in-plane movement over the depth of the laminated composite plates.

Figure 2 shows the variation in in-plane movement over the plate’s thickness at the interfaces between composite layers. The curve represents the displacement configuration at the cross-section of a plate with a general lamination layout based on improved third-order shear deformation theory.

(3) $$\left\{u_{1}\right\}=\left\{u_{1}^{0}\right\}+\sum_{i=0}^{nu-1}S_{1}^{i}(Z-Z_{i})\left\{H\right\}(Z-Z_{i})+\sum_{i=1}^{nl-1}T_{1}^{i}(Z-\rho_{i})\left\{H\right\}(-Z+\rho_{i})+\left\{\xi_{1}\right\}Z^{2}+\left\{\varphi_{1}\right\}Z^{3}$$

(4) $$\left\{u_{2}\right\}=\left\{u_{2}^{0}\right\}+\sum_{i=0}^{nu-1}S_{2}^{i}(Z-Z_{i})\left\{H\right\}(Z-Z_{i})+\sum_{i=1}^{nl-1}T_{2}^{i}(Z-\rho_{i})\left\{H\right\}(-Z+\rho_{i})+\left\{\xi_{2}\right\}Z^{2}+\left\{\varphi_{2}\right\}Z^{3}$$

Equations (3) and (4) represent in-plane displacements in the x and y direction, respectively [42]. Where {*u*^0^} represents the in-plane displacement of any points on the central surface of the plane, and nu and nl represent the number of upper and lower surfaces of the plane. The slopes of the i^th^ layer for upper and lower surfaces are represented by $S_{1}^{i}$, $S_{2}^{i}$, $T_{1}^{i}$, $T_{2}^{i}$ respectively. the unidentified higher-order terms are $\left\{\xi_{1}\right\}$, $\left\{\xi_{1}\right\}$, $\left\{\varphi_{1}\right\}$, $\left\{\varphi_{2}\right\}$. The unit step expressions are $\left\{H\right\}(Z-Z_{i})$, $\left(Z-\rho_{i}\right)$ and the subscripts 1 and 2 denote the coordinate axes (i.e., x,y in these functions).

The lateral displacement is considered to be uniform across the entire thickness of the sheet, i.e.,

(5) $$\left\{u_{3}\right\}=\left\{w\right\}(x,y)$$

By eliminating particular variables from in-plane displacement equations in HZT, zigzag theory is shortened to a third-order concept with the use of the previously mentioned expansion and FSDT. Indeed, expressions (3) and (4) exclude those concepts, making HZT the most common and making HSDT and FSDT its subsets. For the higher deformation concept, $S_{1}^{i}$, $S_{2}^{i}$, $S_{3}^{i},$ $T_{1}^{i}$, $T_{2}^{i}$, $T_{3}^{i}$ are not included, except $S_{1}^{0}$, $T_{1,}^{0\;}S_{2,}^{0}$, $T_{2}^{0},\;\;S_{3}^{0}{,\;T}_{3}^{0}$, and regarding FSDT and anticipating $\xi_{i}$, $\varphi_{i}$, every $S_{\alpha }^{i},\;T_{\alpha }^{i},$ with the exception of $S_{\alpha }^{0}$, $T_{\alpha }^{0},$ where α = 1.2, denotes the coordinate axis of x and y.

Here, by using free boundary conditions and lateral tangential stress at the top and bottom of the plate, $\sigma_{3\alpha /z=\pm h/2}=0$. Now, $\xi_{\alpha }$ and $\varphi_{\alpha }$ of the zigzag theory can be represented as follows, where α = 1.2 represents the *x* and *y* axes.

(6) $$\left\{\varphi_{1}\right\}=-\frac{4}{3h^{2}}\left\{w_{1}+\frac{1}{2}\left(\sum_{i=0}^{nu-1}S_{1}^{i}+\sum_{i=0}^{nl-1}T_{1}^{i}\right)\right\}$$

(7) $$\left\{\varphi_{2}\right\}=-\frac{4}{3h^{2}}\left\{w_{2}+\frac{1}{2}\left(\sum_{i=0}^{nu-1}S_{2}^{i}+\sum_{i=0}^{nl-1}T_{2}^{i}\right)\right\}$$

(8) $$\left\{\xi_{1}\right\}=-\frac{1}{2h}\left\{w_{1}+\frac{1}{2}\left(\sum_{i=0}^{nu-1}S_{1}^{i}-\sum_{i=0}^{nl-1}T_{1}^{i}\right)\right\}$$

(9) $$\left\{\xi_{2}\right\}=-\frac{1}{2h}\left\{w_{2}+\frac{1}{2}\left(\sum_{i=0}^{nu-1}S_{2}^{i}-\sum_{i=0}^{nl-1}T_{2}^{i}\right)\right\}$$

On the other hand, by substituting the lateral tangential stress consistency at the interior layer, the $S_{\alpha \;}and$ $T_{\alpha }$ equations are stated as follows:

(10) $$S_{1}^{i}=a_{1\gamma }^{i}\left(w_{\gamma }+\Psi_{\gamma }\right)+b_{1\gamma }^{i}w{,}_{\gamma }$$

(11) $$S_{2}^{i}=a_{2\gamma }^{i}\left(w_{\gamma }+\Psi_{\gamma }\right)+b_{2\gamma }^{i}w{,}_{\gamma }$$

(12) $$T_{1}^{i}=c_{1\gamma }^{i}\left(w_{\gamma }+\Psi_{\gamma }\right)+d_{1\gamma }^{i}w{,}_{\gamma }$$

(13) $$T_{2}^{i}=c_{2\gamma }^{i}\left(w_{\gamma }+\Psi_{\gamma }\right)+d_{2\gamma }^{i}w{,}_{\gamma }$$

where $a_{1\gamma }^{i}$, $b_{1\gamma }^{i}$, $a_{2\gamma }^{i}$, $b_{2\gamma }^{i}$, $c_{1\gamma }^{i}$, $d_{1\gamma }^{i}$, $c_{2\gamma }^{i}$, $d_{2\gamma }^{i}$ are constants depending on the geometric and material characteristics of individual layers, and $w{,}_{\gamma }$ is the gradient of lateral displacement, where γ = 1.2 and $S_{\alpha }^{0}$ = $\Psi_{\alpha }$ is the angular deformation about perpendicular to the central layer surface of the dimension axis, where (α = 1.2, i.e., *x* and *y* axis). By utilizing expression (1), expressions (3) and (4), and expressions (12) and (13), the strain field vector may be determined; it is represented by

(14) $$\left\{\bar{\varepsilon }\right\}=\left[H\right]\left\{\varepsilon \right\}$$

where $\left\{\bar{\varepsilon }\right\}$ denotes the vector representing the strain field with a 5 × 1 matrix size. At the mid plane, $\left\{\varepsilon \right\}$ represents the modified strain vector with a size of 17 × 1. $\left[H\right]$ is a 5 × 17 matrix representation that includes both terms, including z, and terms pertaining to material qualities.

(15) $${\left\{\varepsilon \right\}}^{T}=\left\{\begin{array}{l} \frac{\delta u_{1}}{\delta x}\frac{\delta u_{2}}{\delta y}\frac{\delta u_{2}}{\delta x}+\frac{\delta u_{1}}{\delta y}\frac{\delta w_{1}}{\delta x}\frac{\delta w_{2}}{\delta y}\frac{\delta w_{2}}{\delta x}\frac{\delta w_{1}}{\delta y}\frac{\delta \psi_{1}}{\delta x}\frac{\delta \psi_{2}}{\delta y}\\ \frac{\delta \psi_{2}}{\delta x}\frac{\delta \psi_{1}}{\delta y}\Psi_{1}\Psi_{2}\frac{\delta w}{\delta x}\frac{\delta w}{\delta y}w_{1}w_{2} \end{array}\right\}$$

(16) $$\left\{\varepsilon \right\}=\left[B\right]\left\{\delta \right\}$$

where $\left[B\right]$, $\left\{\delta \right\}$ denote strain displacement and an unknown nodal vector, having a matrix of size 17 × 63 and 63 × 1, respectively.

### 2.3. Finite Element Formulation

$C^{1}$ consistency to the lateral displacement must be achieved by the displacement fields in order to use the finite element approach. In order to circumvent the issues of C^1^ consistency, the derivatives of w concerning x and y are described as

(17) $$\frac{\delta w}{\delta x}=w_{1}\;\mathrm{and}\;\frac{\delta w}{\delta y}=w_{2}$$

**Figure 3:**
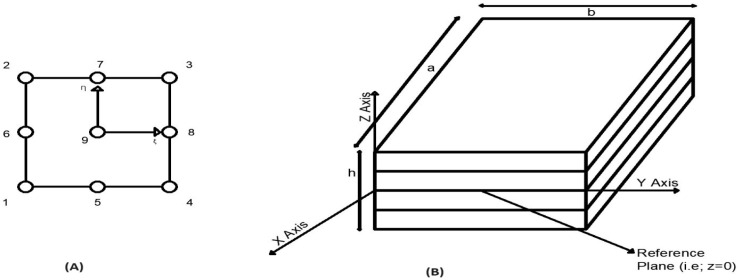
(**A**) Nine noded iso-parametric element. (**B**) Plate with four layers and simple support.

The abovementioned expressions are useful to represent all the unknowns containing $w_{1}$ and $w_{2}$ as $C^{0}$ continuous. In the current investigation, nine noded quadrilateral continuous isoperimetric components with seven degrees of freedom per individual node are applied, as shown in Figure 3.

(18) $$u_{1}=\sum_{i=1}^{9}N_{i}u_{i},\;u_{2}=\sum_{i=1}^{9}N_{i}v_{i},\;u_{3}=\sum_{i=1}^{9}N_{i}u_{i},\;\psi_{1}=\sum_{i=1}^{9}N_{i}\psi_{1i},\;\psi_{2}=\sum_{i=1}^{9}N_{i}\psi_{2i}\;w_{1}=\sum_{i=1}^{9}N_{i}w_{1i},\;w_{2}=\sum_{i=1}^{9}N_{i}w_{2i}$$

where *N_i_* represents the shape function of *i*^th^ node. Shape functions representing the transverse displacement ‘w’ in improved third-order shear deformation theory (ITSDT) are usually selected according to the displacement field expected within the plate. Here, the expressions for the component nodal load vector and element rigidity matrix are derived for the bending analysis. The following element rigidity matrix and equation of equilibrium are created with the use of the Hamilton approach:

(19) $$\left[k^{el}\right]=\sum_{i=1}^{nu+nl}{∭\left[B\right]}^{T}{\left[H\right]}^{T}\left[Q^{-i}\right]\left[H\right]\left[B\right]dxdydz+\left[p_{0}\right]$$

(20) $$\left[k^{el}\right]=\sum_{i=1}^{nu+nl}\iint {\left[B\right]}^{T}\left[D\right]\left[B\right]dxdy+\left[p_{0}\right]$$

where $\left[B\right]$ is the strain matrix, and $\left[Q\right]$ is the transformed material constant matrix. The terms related to material characteristics and the terms containing z constitute the matrix $\left[H\right]$.

Where $\left[D\right]=\int_{k=1}^{n}{\int \left[H\right]}^{T}\left[Q^{-i}\right]\left[H\right]dz$.

Now, using expression (15), the penalty term is defined as

(21) $$\left[p_{0}\right]=\iint \mu (\left\{\frac{\delta w}{\delta x}-w_{1}\right\}\left\{\frac{\delta w}{\delta x}-w_{1}\right\}+\left\{\frac{\delta w}{\delta y}-w_{2}\right\}\left\{\frac{\delta w}{\delta y}-w_{2}\right\})dxdy$$

where μ is the penalty factor.

Expression (18) can be used to provide the component load vector, which can also be generated throughout the computation process.

(22) $$\left[p_{0}\right]={\int \left[N\right]}^{T}qdxdy$$

where q and $\left[N\right]$ are the shape function matrix and the intensity of lateral load, respectively.

### 2.4. Bending Analysis

The format adopted for bending analysis eliminates zeros within the band of the stiffness matrix beyond the last non-zero value and reduces the storage requirement.

The bending equilibrium equation is written as

(23) $$\left[K\right]\left\{\delta \right\}=\left\{P\right\}$$

Equation (23) is solved by the Cholesky decomposition procedure [43]. Efficient techniques, such as an automatic mesh generator and the skyline storage scheme, are incorporated into the in-house computer code. The deflection components {*δ*} at any point of the plate can be calculated by solving the static equilibrium equations, as discussed above. Once these displacements at the reference plane are known, the strain components, {ε}, at any point of the plate can be calculated by using the strain displacement relationship using Equations (15) and (16). The stress is calculated using Equation (1). For the calculation of transverse shear stresses, the respective equilibrium equations are used.

The following equations are used for the calculation of normalized central deflection and stresses for Example 1:

(24) $$\bar{w}=w^{nd}(a/2,b/2),w_{nd}=100wh^{3}E_{2}/qa^{4}$$

(25) $$\bar{\sigma_{1}}=\sigma_{1}^{nd}(a/2,b/2,h/2),\sigma_{1}^{nd}=\frac{h^{2}\sigma_{1}}{qa^{2}}$$

(26) $$\bar{\sigma_{2}}=\sigma_{2}^{nd}(a/2,b/2,h/6),\sigma_{2}^{nd}=\frac{h^{2}\sigma_{2}}{qa^{2}}$$

(27) $$\bar{\sigma_{4}}=\sigma_{4}^{nd}(0,b/2,0),\sigma_{4}^{nd}=\frac{h^{2}\sigma_{4}}{qa^{2}}$$

(28) $$\bar{\sigma_{5}}=\sigma_{5}^{nd}(a/2,0,0),\sigma_{5}^{nd}=\frac{h^{2}\sigma_{5}}{qa^{2}}$$

(29) $$\bar{\sigma_{6}}=\sigma_{1}^{nd}(0,0,h/2),\;\sigma_{6}^{nd}=\frac{h^{2}\sigma_{6}}{qa^{2}}$$

The following formula is used to obtain the normalized deflection and stresses for the remaining examples:

(30) $$\bar{w}=w^{nd}(a/2,a/2,0),w_{nd}=wh^{3}E_{2}/qa^{4}$$

(31) $$\bar{\sigma_{xx}}=\sigma_{xx}^{nd}(a/2,a/2,-h/2),\sigma_{xx}^{nd}=\frac{h^{2}\sigma_{xx}}{qa^{2}}$$

(32) $$\bar{\sigma_{yy}}=\sigma_{yy}^{nd}(a/2,a/2,-h/4),\sigma_{yy}^{nd}=\frac{h^{2}\sigma_{yy}}{qa^{2}}$$

(33) $$\bar{\sigma_{xy}}=\sigma_{xy}^{nd}(0,0,-h/2),\sigma_{xy}^{nd}=\frac{h^{2}\sigma_{xy}}{qa^{2}}$$

(34) $$\bar{\sigma_{xz}}=\sigma_{xz}^{nd}(0,a/2,0),\sigma_{xz}^{nd}=\frac{h\sigma_{xz}}{qa}$$

(35) $$\bar{\sigma_{yz}}=\sigma_{yz}^{nd}(a/2,0,0),\sigma_{yz}^{nd}=\frac{h\sigma_{yz}}{qa}$$

## 3. Results and Discussion

In this work, improved third-order shear deformation theory analysis is performed using a finite element approach. Numerous novel findings are determined regarding the deflection and stresses resulting from modifications in the boundary conditions, orientation angle, length–thickness ratio, modulus ratio, and other factors for the pervious composite plate, as shown in Figure 1A. A 16 × 16 mesh size is used in the calculation of the results. The thickness, density, and orthotropic substance of each layer are taken to be identical. The laminate’s boundary conditions and dimensions are listed as follows: the edges $x_{1}=0,\;a$ and $x_{2}=\pm b/2$ can take any combination of simply supported, clamped (C), and free (F) edge conditions.

**Example** **1.** *In this part, Table 1 presents the results of the analysis of a three-layer rectangular (b/a = 3) cross-ply laminated plate (Figure 1) with a fiber orientation angle of* 0°/90°/0°*, exposed to a sinusoidal load of varying intensity* $q(x,y)=q_{0}\sin(\pi x/a)\sin(\pi y/b)$*and boundary conditions. All the layers have the same material characteristics, even when they are oriented differently* ($E_{1}$ *= 25 GPa,* $E_{2}=1\;GPa$*;* $G_{12}$ *=* $G_{13}$ = 0.5$E_{2}$, $G_{23}$ *= 0.2* $E_{2}$; $\nu_{12}$ *= 0.25 and* $\nu_{13}$ *= 0.01). This research was designed for a thickness ratio of 10 and 100. Table 1 displays the results of the validation and convergence studies. Table 2, Table 3, Table 4 and Table 5 display the new findings.*

Table 1 shows the results of the validation study; the obtained results are compared with those obtained by (Sheikh and Chakrabarti [35], Reddy [36], and Pagano [37]) due to the applied sinusoidal load. Using a three-layer laminated plate with a thickness ratio of 10 to 100, deflection and stresses are examined.

The new results were analyzed for the various boundary conditions, porosity, and orientation angle in Table 2, Table 3, Table 4 and Table 5. In Table 2, deflection and stress are presented in relation to variations in boundary conditions and orientation angle to the fiber in composites using improved third-order theory. For the length-to-thickness ratio of 10 and boundary condition SSSS, deflection is decreased by 8.4% with variation in the fiber orientation angle from 0°/90°/0° to 0°/45°/0°. It also seems that normalized effective stresses ($\bar{\sigma_{1}}$) are also decreased by 5.7% for the same conditions. For the boundary condition CCCC, normalized deflection is decreased by 6.9% with variation in fiber orientation angle from 0°/90°/0° to 0°/60°/0°. Effective stress ($\bar{\sigma_{2}}$) is reduced to 12.8% with variation in fiber orientation angle from 0°/60°/0° to 0°/45°/0°. Due to applied sinusoidal load and SSSC boundary conditions, deflection is decreased by 11.34% and normalized stress ($\bar{\sigma_{3}}$) is increased by 3.8% with variation in orientation angle from 0°/60°/0° to 0°/30°/0°. Here, deflection is also decreased by 5.6% with a variation in orientation angle from 0°/45°/°0° to 0°/30°/°0° with boundary condition SSCC and a thickness ratio of 10. It helps to have a lightweight structure. It seems that normalized stress ($\bar{\sigma_{4}}$) is also decreased by 14.5% for the same conditions. For the FFCC boundary condition and variation in fiber orientation angle from 0°/60°/0° to 0°/45°/0°, deflection is reduced by 6% and normalized stress ($\bar{\sigma_{6}}$) is also reduced by 11.3%.

In Table 3, deflection and stresses are examined after the application of a porosity distribution of 0.1 throughout the plate thickness. A porosity distribution of 0.1 and SSSS boundary condition, normalized deflection is decreased by 4.4% as compared to without porosity effects, with a fiber orientation angle of 0°/90°/0° and a thickness ratio of 10. In Table 3, considering sinusoidal load and porosity effects, deflection is reduced by 14% with variation in the orientation angle from 0°/90°/0° to 0°/45°/0° and with boundary condition CCCC. Here, normalized stress ($\bar{\sigma_{1}}$) is also reduced by 9.6% for the same boundary conditions.

In Table 3, for the boundary conditions of SSSC, normalized deflection is reduced to 4.8% with a porosity of 0.1 as compared to negligible porosity. It is further reduced by 6.5% with a change in orientation angle from 0°/90°/0° to 0°/60°/0°. For the length-to-thickness ratio of 10 and FFCC boundary conditions, normalized stress ($\bar{\sigma_{2}}$) is decreased by 3% with a change in orientation angle from 0°/90°/0° to 0°/30°/0°. With a porosity distribution of 0.1 and boundary condition SSCC, normalized stress ($\bar{\sigma_{3}}$) is decreased by 2.2% with a change in orientation angle from 0°/90°/0° to 0°/60°/0°. For the boundary condition FFCC, deflection is decreased by 4.7% with a porosity of 0.1, as compared to negligible porosity, as specified in Table 2.

In Table 4, a porosity distribution of 0.2 is applied, further taking into account normalized stresses and deflection under various boundary conditions and a thickness ratio of 10. For the boundary condition SSSS and orientation angles of 0°/90°/0°, normalized deflection is reduced by 5.6% with a porosity allocation of 0.2, as compared to a porosity of 0.1, as specified in Table 3. With a porosity distribution of 0.2 and boundary condition CCCC, normalized effective stress ($\bar{\sigma_{1}}$) is decreased by 13.3% when the orientation angle varies from 0°/90°/0° to 0°/30°/0°. Here, normalized stress ($\bar{\sigma_{3}}$) is increased by 11.9% with a shift in orientation angle from 0°/90°/0° to 0°/60°/0° and having all fixed edges. Deflection is decreased by 11.3% due to change in orientation angle from 0°/90°/0° to 0°/45°/0° with a porosity effect of 0.2 and boundary condition SSSC. For a thickness ratio of 10 and boundary condition FFCC, normalized stress ($\bar{\sigma_{3}}$) is decreased by 2.5% with a shift in orientation angle from 0°/90°/0° to 0°/30°/0°. With variation in the fiber orientation angle from 0°/90°/0° to 0°/45°/0°, normalized effective stress ($\bar{\sigma_{4}}$) is decreased by 14.6% under boundary condition SSCC. For the orientation angle of 0°/90°/0°, deflection is decreased by 5% with a porosity of 0.2, as compared to a porosity value of 0.1, as specified in Table 3. Here, stress ($\bar{\sigma_{6}}$) is also decreased by 2% with a shift in fiber orientation angle from 0°/60°/0° to 0°/30°/0°, having a porosity value of 0.2 and a length–thickness ratio of 10.

In Table 5, a sinusoidal load is applied along with a porosity value of 0.3, applied to the whole thickness of the sheet. For the SSSS boundary condition, normalized deflection is decreased by 5.4%, as compared to the deflection mentioned in Table 4 for a fiber orientation angle of 0°/90°/0°. Here, normalized effective stress ($\bar{\sigma_{1}}$) is also reduced to 6.7% with a change in fiber orientation angle from 0°/90°/0° to 0°/30°/0°. For a thickness ratio of 10 and the CCCC boundary condition, normalized deflection is decreased by 14% with a shift in orientation angle from 0°/90°/0° to 0°/45°/0° and having porosity of 0.3. It is also observed that effective stress ($\bar{\sigma_{2}}$) is reduced by 8% with a shift in orientation to the fiber from 0°/60°/0° to 0°/30°/0°, having the same boundary conditions. Normalized deflection is decreased by 10.5% with variation in fiber orientation angle from 0°/90°/0° to 0°/45°/0° and with boundary condition SSSC. In the current study, stress ($\bar{\sigma_{3}}$) is increased by 4% with variation in orientation to the fiber from 0°/90°/0° to 0°/30°/0°. In this case, for a porosity effect of 0.3 and the FFCC boundary condition, normalized deflection and stress ($\bar{\sigma_{4}}$) are decreased by 14.9% and 10.9% with shift in orientation angle of 0°/90°/0° to 0°/30°/0°, respectively. For the SSCC boundary condition of the sheet, it is found that deflection is reduced by 6.5% and stress is increased by 8% with a variation in the orientation angle from 0°/90°/0° to 0°/60°/0°. Considering boundary condition FFCC and a length–thickness ratio of 10, normalized deflection is reduced by 9.6% with a shift in orientation angle from 0°/90°/0° to 0°/30°/0°, with porosity value of 0.3.

**Example** **2.** *For the purpose of bending analysis of the laminated pervious sheet, the following analytical problem is solved. The authors considered a four-layer, square (a = b), laminated plate, resting on simply supported end conditions. The orientation angle was* 0°*/*90°*/*90°/0°*, and material properties were as follows:*$\;E_{1}$*= 175,* $E_{2}$*= 7,* $G_{12}$*=* $G_{13}$*= 3.5,* $G_{23}$*= 1.4,* $\upsilon_{12}$*= 0.25, and a/h = 10. A sinusoidal loading pattern is applied, with* $q(x,y)=q_{0}\sin(\pi x/a)\sin(\pi y/b)$*. Table 6 presents a validation analysis for the stresses and deflections of the square layered plate under a sinusoidal load with different boundary conditions. Table 7, Table 8, Table 9 and Table 10 present new findings that are examined with varying porosity patterns, boundary conditions, fiber orientations, material parameters, etc.*

Table 6 shows the results of the validation analysis for the different material properties; the obtained results are compared with those reported by (Tasneem et al. [38]) with regard to applied sinusoidal load. Deflection and stresses were analyzed at various points with the four-layer composite plate having thickness ratio of 10.

New results were analyzed for various boundary conditions, porosity, material properties, and orientation angles, as presented in Table 7, Table 8, Table 9 and Table 10. In Table 7, deflection and stresses at different points are examined with variation in edge conditions and orientation angle to the fiber in composites using improved third-order theory. The adjustment in orientation angle from 0°/90°/90°/0° to 0°/60°/60°/0° reduces the deflection by 6.9% for a thickness ratio of 10 and a boundary condition of SSSS. It also seems that normalized effective stress ($\bar{\sigma_{xx}}$) is decreased by 6.6% for an orientation angle change from 0°/90°/90°/0° to 0°/60°/60°/0°. For the boundary condition CCCC, normalized deflection is decreased by 8% with a change in the fiber orientation angle from 0°/90°/90°/0° to 0°/30°/30°/0°. Effective stress ($\bar{\sigma_{yy}}$) is decreased by 44.4% with a variation in orientation angle from 0°/60°/60°/0° to 0°/45°/45°/0°. For an applied sinusoidal load and the SSSC boundary condition, deflection is decreased by 1.5% and normalized stress ($\bar{\sigma_{xy}}$) is increased by 23.6% with an adjustment in the fiber orientation angle from 0°/90°/90°/0° to 0°/45°/45°/0° and 0°/90°/90°/0°to 0°/30°/30°/0° , respectively. Here, normalized stress ($\bar{\sigma_{xz}}$) is increased by 15% when the orientation angle is changed from 0°/60°/60°/0° to 0°/45°/45°/0° with boundary condition SSCC and a thickness ratio of 10.

A porosity distribution of 0.1 is applied over the whole depth of the plate and results in effects on the deflection and stresses, as specified in Table 8. For a porosity distribution of 0.1 and the SSSS boundary condition, normalized deflection is decreased by 4.5% as compared to the case without porosity effects, having a fiber orientation of 0°/90°/90°/0° and a thickness ratio of 10. As shown in Table 8, considering sinusoidal load and porosity effects, deflection is reduced by 12.1% with a variation in orientation angle from 0°/90°/90°/0° to 0°/45°/45°/0° for boundary condition CCCC. Here, normalized stress ($\bar{\sigma_{xx}}$) is also reduced by 6.5% for the same boundary conditions. As shown in Table 8, for the boundary condition SSSC, normalized deflection is decreased by 6% with a porosity of 0.1, compared to negligible porosity, for the orientation angle of 0°/90°/90°/0°. For the length-to-thickness ratio of 10 and the FFCC boundary condition, normalized stress ($\bar{\sigma_{yy}}$) is reduced by 14.71% with variation in orientation angle from 0°/90°/90°/0° to 0°/60°/60°/0°. For a porosity distribution of 0.1 and boundary condition SSCC, normalized stress ($\bar{\sigma_{xy}}$) is reduced by 16.5% with variation in the fiber orientation from 0°/60°/60°/0° to 0°/45°/45°/0°. For the boundary condition FFCC, deflection is decreased by 1.5% for a porosity of 0.1, as compared to negligible porosity, for the orientation angle of 0°/60°/60°/0°, as specified in Table 7.

Table 9 shows the results for the application of a porosity distribution of 0.2, taking into account normalized stresses and deflection under various boundary conditions and a thickness ratio of 10. For the boundary condition SSSS and an orientation angle of 0°/90°/90°/0°, normalized deflection is reduced by 6.5% for a porosity distribution of 0.2, as compared to a porosity of 0.1, as specified in Table 8. With a porosity distribution of 0.2 and boundary condition CCCC, normalized effective stress ($\bar{\sigma_{xx}}$) is increased by 4.3% with a change in orientation angle from 0°/90°/90°/0° to 0°/45°/45°/0°. Here, normalized stress ($\bar{\sigma_{xy}}$) is increased by 13.2% with a variation in orientation angle from 0°/60°/60°/0° to 0°/45°/45°/0° with all edges fixed. Deflection is decreased by 5.1% with a variation in fiber orientation from 0°/45°/45°/0° to 0°/30°/30°/0° and with a porosity effect of 0.2 and boundary condition SSSC. For a length-to-thickness ratio of 10 and boundary condition FFCC, normalized stresses ($\bar{\sigma_{xy}}$) is increased to 17.88% by changing orientation the angle from 0°/60°/60°/0° to 0°/45°/45°/0°. With a variation in the orientation angle from 0°/90°/90°/0° to 0°/60°/60°/0°, normalized effective stress ($\bar{\sigma_{xz}}$) is decreased by 26.58% under boundary condition SSCC. For the orientation angle of 0°/90°/90°/0°, deflection is decreased by 1.2% for a porosity of 0.2, as compared to porosity value of 0.1, under the FFFC boundary condition, as specified in Table 8. Here, normalized stress ($\bar{\sigma_{yz}}$) is also decreased by 1.8% with a variation in the orientation angle of 0°/60°/60°/0° to 0°/45°/45°/0°, with a porosity distribution of 0.2 and a thickness ratio of 10.

Table 10 specifies the results for a sinusoidal load applied along with a porosity allocation of 0.3 in the whole depth of the plate. For the SSSS boundary condition, normalized deflection is decreased by 5.5%, as compared to the deflection mentioned in Table 9 for an orientation angle of 0°/90°/90°/0°. Here, normalized stress ($\bar{\sigma_{xx}}$) is also decreased by 6.3% due to a change in the fiber orientation from 0°/90°/90°/0° to 0°/45°/45°/0°. For a thickness ratio of 10 and the CCCC boundary condition, normalized deflection is reduced by 8.3% with variation of the applied orientation angle from 0°/90°/90°/0° to 0°/30°/30°/0°, with a porosity of 0.3. It is also observed that effective stress ($\bar{\sigma_{yy}}$) is reduced by 35.9% with variation in the orientation angle from 0°/90°/90°/0° to 0°/60°/60°/0° under the same boundary conditions. Normalized deflection is reduced to 1.2% with a change in the orientation angle from 0°/90°/90°/0° to 0°/45°/45°/0° under boundary condition SSSC. For the same boundary conditions, stress ($\bar{\sigma_{xy}}$) is reduced to 4.8% with variation in the orientation angle from 0°/60°/60°/0° to 0°/45°/45°/0°. For a porosity value of 0.3 and the FFCC boundary condition, normalized stress ($\bar{\sigma_{xz}}$) is decreased by 33.3% with variation in the orientation angle from 0°/60°/60°/0° to 0°/45°/45°/0°.

For the SSCC boundary condition of the plate, it was observed that stress is increased by 25.75% with variation in the orientation angle from 0°/90°/90°/0° to 0°/60°/60°/0°. Considering boundary condition FFCC and a thickness ratio of 10, normalized stress ($\bar{\sigma_{yz}}$) is reduced by 2.3% with variation in the orientation angle from 0°/60°/60°/0° to 0°/45°/45°/0° under a porosity value of 0.3.

**Example** **3.** *This example covers the subjecting of a two-layer, square, cross-ply laminate with an orientation angle of 0°/90° to a sinusoidal load of varying intensity of* $q(x,y)=q_{0}\sin(\pi x/a)\sin(\pi y/b)$ *under various boundary conditions. Even though the orientation of each layer varies, the material characteristics are the same in every layer* ($E_{1}$ *= 25 GPa,* $E_{2}=1\;GPa$*;* $G_{12}$ *=* $G_{13}$ *= 0.5* $E_{2}$*,* $G_{23}$ *= 0.2* $E_{2}$*;* $\nu_{12}$ *= 0.25 and* $\nu_{13}$ *= 0.01). This study is conducted for a thickness ratio of 10. Results of the validation study are shown in Table 11. In Table 12, new findings are presented. The normalized central deflection is calculated using Equation (24).*

Table 11 shows the results of a validation study performed for a two-layer antisymmetric cross-ply laminated plate under sinusoidal loading with a thickness ratio of 10. The work was compared with existing studies (Liu et al. [4], Khdeir and Reddy [48], and Vel and Batra [49]). In Table 12, new results are presented under various porosity, orientation angle, and boundary conditions. The applied porosity values amounted to 0, 0.1, 0.2, and 0.3.

Table 12 shows the results for a sinusoidal load applied along with the porosity values of 0.1, 0.2, and 0.3 in the whole depth of the plate for a thickness ratio of 10. For the SSSS boundary condition and a porosity of 0.1, normalized deflection is reduced by 11.7%, as compared to negligible porosity under an orientation angle of 0°/90°. Deflection is further reduced by 16% with variation in fiber orientation from 0°/90° to 0°/60°. For a thickness ratio of 10 and the SSCC boundary condition, normalized deflection is increased by 19.2% with variation in the applied orientation angle from 0°/90° to 0°/30° and having porosity of 0.1. Normalized deflection is reduced to 25.35% when the orientation angle is changed from 0°/90° to 0°/45°, under boundary condition SSFF and a porosity of 0.2. For the porosity effect of 0.2 and the SSSC boundary condition, normalized deflection is decreased by 11.2% with variation in the orientation angle from 0°/90° to 0°/30°/45°. For a thickness ratio of 10 and the SSFS boundary condition, normalized deflection is reduced by 12.5% with variation in the applied orientation angle from 0°/60° to 0°/45°, with a porosity of 0.2. Normalized deflection is reduced to 26.3% when the orientation angle changes from 0°/90° to 0°/45°, with boundary condition SSSS and a porosity of 0.3. For the porosity effect of 0.3 and the SSFC boundary condition, normalized deflection is decreased by 4.2% with variation in the orientation angle from 0°/60° to 0°/45°.

**Example** **4.** *This example covers subjecting a three-layer, square, cross-ply laminate with an orientation angle of* 0°*/*90°*/*0° *to a sinusoidal load of varying intensity of* $q(x,y)=q_{0}\sin(\pi x/a)\sin(\pi y/b)$ *under various boundary conditions. Even though the orientation of each layer varies, the material characteristics are the same in every layer* ($E_{1}$ *= 25 GPa,* $E_{2}=1\;GPa$*;* $G_{12}$ *=* $G_{13}$ *= 0.5* $E_{2}$*,* $G_{23\;}$ *= 0.2* $E_{2}$*;* $\nu_{12}$ *= 0.25 and* $\nu_{13\;}$ *= 0.01). Results of the validation study are demonstrated in Table 13 for thickness ratios of 5 and 10. New results are given in* Table 12 and Table 13 *for the same thickness ratios. The normalized central deflection was calculated using Equation (24).*

Table 13 shows the results of a validation study performed for three-layer cross-ply laminated plate under sinusoidal loading with a thickness ratio of 10. The work was compared with existing studies (Liu et al. [5] Vel and Batra [49]). New results were calculated with the help of in-house FOTRAN code under various porosity, orientation angle, and boundary conditions, as specified in Table 14 and Table 15. The porosity values of 0, 0.1, 0.2, and 0.3 were considered.

Table 14 shows the results for a sinusoidal load applied along with the porosity values of 0.1, 0.2, and 0.3 over the whole depth of the plate having thickness ratio of 5. For the SSSS boundary condition and a porosity of 0.1, normalized deflection is reduced by 6.5%, as compared to negligible porosity, with orientation angle of 0°/90°/0°. Deflection is further reduced by 19% with variation in fiber orientation from 0°/90°/0° to 0°/45°/0°. For a thickness ratio of 10 and the SSCC boundary condition, normalized deflection is increased by 16.5% with the applied orientation angle changed from 0°/90°/0° to 0°/30°/0° and having a porosity of 0.1. Normalized deflection is reduced to 19.4% when the orientation angle changes from 0°/90°/0° to 0°/45°/0°, under boundary condition SSFF and a porosity of 0.1. For the porosity effect of 0.2 and the SSSC boundary condition, normalized deflection is decreased by 5.6% by variation in the orientation angle from 0°/60°/0° to 0°/45°/0°. For the thickness ratio of 5 and the SSFS boundary condition, normalized deflection is reduced by 21.8% with an applied orientation angle change from 0°/60°/0° to 0°/45°/0°, under a porosity of 0.2. Normalized deflection is reduced to 17.3% when the orientation angle changes from 0°/90°/0° to 0°/45°/0°, with boundary condition SSSS and a porosity of 0.3. For the porosity effect of 0.3 and the SSFC boundary condition, normalized deflection is reduced by 14.9% with variation in the orientation angle from 0°/90°/0° to 0°/60°/0°.

Table 15 shows the results for a sinusoidal load applied along with the porosity values of 0.1, 0.2, and 0.3 over the whole depth of the plate having thickness ratio of 10. For the SSSS boundary condition and a porosity of 0.1, normalized deflection is reduced by 4.8%, as compared to negligible porosity, with an orientation angle of 0°/90°/0°. Deflection is further reduced by 12.3% with variation in fiber orientation from 0°/90°/0° to 0°/30°/0°. For a thickness ratio of 10 and the SSCC boundary condition, normalized deflection is increased by 10.4% with an applied orientation angle change from 0°/90°/0° to 0°/45°/0° and with a porosity of 0.1. Normalized deflection is reduced to 27.2% when the orientation angle changes from 0°/90°/0° to 0°/60°/0°, under boundary condition SSFF and a porosity of 0.2. For the porosity value of 0.2 and the SSSC boundary condition, normalized deflection is increased by 8.8% by changing the orientation angle from 0°/60°/0° to 0°/45°/0°. For a thickness ratio of 10 and the SSFS boundary condition, normalized deflection is reduced by 27.2% with an applied orientation angle change from 0°/60°/0° to 0°/45°/0°, with a porosity of 0.2. Normalized deflection is reduced to 9% when the orientation angle changes from 0°/90°/0° to 0°/30°/0°, under boundary condition SSSS and a porosity of 0.3. For the porosity effect of 0.3 and the SSFC boundary condition, normalized deflection is decreased by 14.9% with variation in the orientation angle from 0°/90°/0° to 0°/60°/0°.

## 4. Conclusions

Improved third-order deformation theory was used throughout the study to examine the bending of laminated porous composite plates. The analysis included consideration of various boundary conditions, material attributes, orientation angles, length-to-width ratios, and modulus ratios. The primary findings for the current study are as follows:

- By increasing the porosity dispersion over the laminated plate thickness, such as at *p* = 0, 0.1, 0.2, and 0.3, the normalized deflection is reduced.
- The bending performance of the layered plate is significantly influenced by the angle at which the fibers are oriented in the composite
- Normalized deflection is reduced at the orientation angle of 0°/90°/0° as the length–thickness ratio of the sheet increases.
- In most boundary condition scenarios, the normalized deflection reduces along with orientation angle change from 0°/90°/90°/0° to 0°/30°/30°/0°.
- As was shown in this study, material characteristics also affect deflection and stresses.
- According to the current research, adding porosities causes a relatively greater change in deflection than stress, thereby aiding in the development of lightweight constructions.
- Some of the potential research areas in this fascinating field, including thermoelastic characteristics, viscoelastic characteristics, and thermomechanical stress, can be further explored using ITSDT evaluation to maximize the performance of the suggested laminated composite pervious plate and shell construction.

## Figures and Tables

**Table 1:** Validation study of three-layer laminated plate.

a/h	Reference	Theory	$\bar{w}$	$\bar{\sigma_{1}}$	$\bar{\sigma_{2}}$	$\bar{\sigma_{3}}$	$\bar{\sigma_{4}}$	$\bar{\sigma_{6}}$
10	Present study (16 × 16)	ITSDT	0.9587	0.7527	0.0439	0.3587	0.0119	0.0125
	Sheikh and Chakrabarti [44]	HSDT	0.8649	0.7164	0.0383	0.2851	0.0106	0.0117
		FSDT	0.8013	0.6398	0.0367	0.1861	0.0110	0.0103
	Reddy [45]	HSDT	0.8622	0.6924	0.0398	0.2859	0.0170	0.0115
		FSDT	0.8030	0.6214	0.0375	0.1894	0.0159	0.0105
	Pagano [46]	3D-Elasticity	0.9190	0.7250	0.0435	0.4200	0.0152	0.0123
100	Present study (16 × 16)	ITSDT	0.5058	0.6706	0.0242	0.3690	0.0084	0.0087
	Sheikh and Chakrabarti [44]	HSDT	0.5097	0.6457	0.0253	0.2847	0.0129	0.0085
		FSDT	0.5091	0.6449	0.0252	0.1866	0.0127	0.0084
	Reddy [45]	HSDT	0.5070	0.6240	0.0253	0.2886	0.0129	0.0083
		FSDT	0.5064	0.6233	0.0253	0.1897	0.0127	0.0083
	Pagano [46]	3D-Elasticity	0.5080	0.6240	0.0253	0.4390	0.0108	0.0083

**Table 2:** New results for three-layer laminated plate.

Boundary Conditions	Reference (Theory)	Orientation Angle	$\bar{w}$	$\bar{\sigma_{1}}$	$\bar{\sigma_{2}}$	$\bar{\sigma_{3}}$	$\bar{\sigma_{4}}$	$\bar{\sigma_{6}}$
SSSS	Present study (ITSDT)	0°/90°/0°	0.9587	0.7527	0.0439	0.3587	0.0119	0.0125
		0°/60°/0°	0.8923	0.7308	0.0671	0.3521	0.0129	0.0135
		0°/45°/°0°	0.8385	0.7099	0.0637	0.3542	0.0128	0.0126
		0°/30°/0°	0.7913	0.6901	0.0428	0.3657	0.0114	0.0119
CCCC	Present study (ITSDT)	0°/90°/0°	0.4827	0.3605	0.0208	0.1222	0.0173	0.0014
		0°/60°/0°	0.4494	0.3423	0.0319	0.1379	0.0158	0.0019
		0°/45°/°0°	0.4147	0.3240	0.0278	0.1548	0.0137	0.0044
		0°/30°/0°	0.3787	0.3065	0.0152	0.1796	0.0113	0.0019
SSSC	Present study (ITSDT)	0°/90°/0°	0.9594	0.7532	0.0436	0.3590	0.0119	0.0125
		0°/60°/0°	0.8927	0.7311	0.0669	0.3522	0.0129	0.0135
		0°/45°/°0°	0.8386	0.7101	0.0636	0.3542	0.0128	0.0127
		0°/30°/0°	0.7914	0.6902	0.0402	0.3657	0.0113	0.0119
FFCC	Present study (ITSDT)	0°/90°/0°	0.9594	0.7532	0.0438	0.3590	0.0049	0.0090
		0°/60°/0°	0.8926	0.7310	0.0676	0.3522	0.0048	0.0106
		0°/45°/°0°	0.8386	0.7096	0.0646	0.3543	0.0048	0.0094
		0°/30°/0°	0.7914	0.6893	0.0409	0.3659	0.0044	0.0085
SSCC	Present study(ITSDT)	0°/90°/0°	0.9602	0.7538	0.0432	0.3593	0.0285	0.0066
		0°/60°/0°	0.8929	0.7314	0.0666	0.3523	0.0268	0.0073
		0°/45°/°0°	0.8387	0.7103	0.0635	0.3542	0.0241	0.0072
		0°/30°/0°	0.7914	0.6903	0.0402	0.3657	0.0206	0.0070
FFCC	Present study (ITSDT)	0°/90°/0°	0.9587	0.7527	0.0441	0.3587	0.0049	0.0090
		0°/60°/0°	0.8923	0.7307	0.0676	0.3521	0.0048	0.0106
		0°/45°/°0°	0.8385	0.7095	0.0646	0.3543	0.0047	0.0094
		0°/30°/0°	0.7914	0.6892	0.0409	0.3659	0.0044	0.0085

**Table 3:** New results for three-layer laminated porous plate (*p* = 0.1).

Boundary Conditions	Reference (Theory)	Orientation Angle	$\bar{w}$	$\bar{\sigma_{1}}$	$\bar{\sigma_{2}}$	$\bar{\sigma_{3}}$	$\bar{\sigma_{4}}$	$\bar{\sigma_{6}}$
SSSS	Present study (ITSDT)	0°/90°/0°	0.9171	0.7392	0.0420	0.3597	0.0125	0.0134
		0°/60°/0°	0.8523	0.7192	0.0635	0.3519	0.0134	0.0143
		0°/45°/°0°	0.8042	0.6998	0.0627	0.3538	0.0128	0.0135
		0°/30°/0°	0.7610	0.6814	0.0401	0.3655	0.0118	0.0127
CCCC	Present study (ITSDT)	0°/90°/0°	0.4498	0.3477	0.0195	0.1265	0.0168	0.0015
		0°/60°/0°	0.4186	0.3311	0.0305	0.1422	0.0154	0.0020
		0°/45°/°0°	0.3863	0.3143	0.0271	0.1589	0.0133	0.0021
		0°/30°/0°	0.3529	0.2983	0.0152	0.1758	0.0111	0.0020
SSSC	Present study (ITSDT)	0°/90°/0°	0.9135	0.7398	0.0417	0.3599	0.0125	0.0134
		0°/60°/0°	0.8536	0.7194	0.0651	0.3520	0.0134	0.0143
		0°/45°/°0°	0.8043	0.7000	0.0626	0.3539	0.0132	0.0135
		0°/30°/0°	0.7611	0.6815	0.0400	0.3656	0.0118	0.0127
FFCC	Present study (ITSDT)	0°/90°/0°	0.9135	0.7398	0.0419	0.3599	0.0050	0.0094
		0°/60°/0°	0.8536	0.7193	0.0658	0.3521	0.0049	0.0110
		0°/45°/°0°	0.8044	0.6996	0.0635	0.3540	0.0049	0.0098
		0°/30°/0°	0.7611	0.6807	0.0406	0.3658	0.0046	0.0089
SSCC	Present study (ITSDT)	0°/90°/0°	0.9144	0.7403	0.0413	0.3602	0.0283	0.0072
		0°/60°/0°	0.8539	0.7197	0.0649	0.3521	0.0267	0.0079
		0°/45°/°0°	0.8045	0.7001	0.0616	0.3539	0.0240	0.0078
		0°/30°/0°	0.7612	0.6816	0.0400	0.3656	0.0206	0.0075
FFCC	Present study (ITSDT)	0°/90°/0°	0.9135	0.7393	0.0422	0.3597	0.0051	0.0094
		0°/60°/0°	0.8534	0.7191	0.0658	0.3519	0.0049	0.0110
		0°/45°/°0°	0.8042	0.6995	0.0636	0.3539	0.0049	0.0098
		0°/30°/0°	0.7610	0.6806	0.0406	0.3658	0.0045	0.0089

**Table 4:** New results for three-layer laminated porous plate (*p* = 0.2).

Boundary Conditions	Reference (Theory)	Orientation Angle	$\bar{w}$	$\bar{\sigma_{1}}$	$\bar{\sigma_{2}}$	$\bar{\sigma_{3}}$	$\bar{\sigma_{4}}$	$\bar{\sigma_{6}}$
SSSS	Present study (ITSDT)	0°/90°/0°	0.8681	0.7256	0.0410	0.3606	0.0132	0.0145
		0°/60°/0°	0.8136	0.7037	0.0635	0.3517	0.0140	0.0153
		0°/45°/°0°	0.7697	0.6898	0.0616	0.3534	0.0138	0.0145
		0°/30°/0°	0.7305	0.6726	0.0397	0.3653	0.0125	0.0138
CCCC	Present study (ITSDT)	0°/90°/0°	0.4161	0.3347	0.0182	0.1313	0.0163	0.0016
		0°/60°/0°	0.3873	0.3197	0.0175	0.1469	0.0149	0.0022
		0°/45°/°0°	0.3576	0.3046	0.0263	0.1634	0.0130	0.0022
		0°/30°/0°	0.3269	0.2902	0.0256	0.1800	0.0108	0.0021
SSSC	Present study (ITSDT)	0°/90°/0°	0.8685	0.7261	0.0398	0.3608	0.0132	0.0145
		0°/60°/0°	0.8136	0.7075	0.0633	0.3517	0.0140	0.0154
		0°/45°/°0°	0.7698	0.6897	0.0615	0.3535	0.0138	0.0145
		0°/30°/0°	0.7306	0.6726	0.0397	0.3654	0.0124	0.0138
FFCC	Present study (ITSDT)	0°/90°/0°	0.8680	0.7261	0.0400	0.3608	0.0053	0.0100
		0°/60°/0°	0.8135	0.7075	0.0638	0.3517	0.0051	0.0115
		0°/45°/°0°	0.7698	0.6894	0.0623	0.3535	0.0052	0.0103
		0°/30°/0°	0.7306	0.6720	0.0402	0.3656	0.0047	0.0094
SSCC	Present study (ITSDT)	0°/90°/0°	0.8688	0.7265	0.0395	0.3611	0.0281	0.0078
		0°/60°/0°	0.8137	0.7078	0.0631	0.3518	0.0265	0.0085
		0°/45°/°0°	0.7699	0.6899	0.0615	0.3535	0.0240	0.0084
		0°/30°/0°	0.7306	0.6727	0.0397	0.3654	0.0208	0.0083
FFCC	Present study (ITSDT)	0°/90°/0°	0.8680	0.7256	0.0403	0.3606	0.0053	0.0100
		0°/60°/0°	0.8136	0.7073	0.0639	0.3517	0.0051	0.0115
		0°/45°/°0°	0.7697	0.6893	0.0623	0.3535	0.0050	0.0103
		0°/30°/0°	0.7305	0.6719	0.0402	0.3655	0.0047	0.0094

**Table 5:** New results for three-layer laminated porous plate (*p* = 0.3).

Boundary Conditions	Reference (Theory)	Orientation Angle	$\bar{w}$	$\bar{\sigma_{1}}$	$\bar{\sigma_{2}}$	$\bar{\sigma_{3}}$	$\bar{\sigma_{4}}$	$\bar{\sigma_{6}}$
SSSS	Present study (ITSDT)	0°/90°/0°	0.8218	0.7117	0.0381	0.3615	0.0141	0.0159
		0°/60°/0°	0.7742	0.6952	0.0615	0.3513	0.0148	0.0166
		0°/45°/°0°	0.7349	0.6791	0.0604	0.3529	0.0146	0.0158
		0°/30°/0°	0.6993	0.6635	0.0394	0.3651	0.0132	0.0151
CCCC	Present study (ITSDT)	0°/90°/0°	0.3815	0.3215	0.0168	0.1368	0.0157	0.0017
		0°/60°/0°	0.3553	0.3083	0.0276	0.1521	0.0145	0.0023
		0°/45°/°0°	0.3282	0.2943	0.0254	0.1684	0.0127	0.0024
		0°/30°/0°	0.3006	0.2819	0.0252	0.1846	0.0106	0.0022
SSSC	Present study (ITSDT)	0°/90°/0°	0.8218	0.7121	0.0378	0.3616	0.0141	0.0159
		0°/60°/0°	0.7742	0.6954	0.0613	0.3513	0.0148	0.0167
		0°/45°/°0°	0.7350	0.6792	0.0603	0.3529	0.0146	0.0158
		0°/30°/0°	0.6998	0.6636	0.0393	0.3651	0.0132	0.0151
FFCC	Present study (ITSDT)	0°/90°/0°	0.8225	0.7121	0.0381	0.3617	0.0055	0.0106
		0°/60°/0°	0.7742	0.6954	0.0618	0.3513	0.0053	0.0121
		0°/45°/°0°	0.7349	0.6789	0.0609	0.3530	0.0052	0.0109
		0°/30°/0°	0.6997	0.6630	0.0397	0.3652	0.0049	0.0100
SSCC	Present study (ITSDT)	0°/90°/0°	0.8225	0.7125	0.0375	0.3618	0.0279	0.0087
		0°/60°/0°	0.7743	0.6956	0.0612	0.3514	0.0265	0.0094
		0°/45°/°0°	0.7350	0.6793	0.0602	0.3530	0.0241	0.0093
		0°/30°/0°	0.6998	0.6636	0.0393	0.3651	0.0210	0.0091
FFCC	Present study (ITSDT)	0°/90°/0°	0.8218	0.7117	0.0383	0.3615	0.0056	0.0106
		0°/60°/0°	0.7742	0.6952	0.0618	0.3512	0.0053	0.0121
		0°/45°/°0°	0.7748	0.6788	0.0609	0.3530	0.0052	0.0109
		0°/30°/0°	0.6997	0.6630	0.0397	0.3652	0.0049	0.0100

**Table 6:** Validation analysis with different boundary conditions of normalized deflection and stresses for the square laminate with orientation angle 0°/90°/90°/0°.

Boundary Conditions	Reference	Theory	$\bar{w}$ × 10^2^	$\bar{\sigma_{xx}}$	$\bar{\sigma_{yy}}$	$\bar{\sigma_{xy}}$	$\bar{\sigma_{xz}}$	$\bar{\sigma_{yz}}$
SSSS	Present study (16 × 16)	ITSDT	0.762	0.556	0.417	0.0270	0.256	0.122
	Tasneem et al. [47]	HSDT	0.719	0.570	0.397	0.0276	0.277	0.156
CCCC	Present study (16 × 16)	ITSDT	0.37247	0.23406	0.2502	0.00138	0.0932	0.1170
	Tasneem et al. [47]	HSDT	0.36119	0.24582	0.2433	0.00230	0.1913	0.2285
SSSC	Present study (16 × 16)	ITSDT	0.6230	0.4542	0.3786	0.0241	0.2108	0.1261
	Tasneem et al. [47]	HSDT	0.5974	0.4668	0.3553	0.0251	0.2383	0.1625
FFCC	Present study (16 × 16)	ITSDT	0.6987	0.1491	0.4889	0.00204	0.0271	0.2017
	Tasneem et al. [47]	HSDT	0.7157	0.1527	0.4961	0.00263	0.0342	0.4225
SSCC	Present study (16 × 16)	ITSDT	0.5108	0.3569	0.3474	0.00655	0.1738	0.1567
	Tasneem et al. [47]	ITSDT	0.4977	0.3932	0.3377	0.00808	0.1926	0.3106
FFFC	Present study (16 × 16)	ITSDT	5.828	0.15717	0.6419	0.00079	0.0254	0.00102
	Tasneem et al. [47]	HSDT	5.860	0.16059	0.7381	0.00098	0.0343	0.00138

**Table 7:** New results for four-layer laminated plate.

Boundary Conditions	Reference (Theory)	Orientation Angle	$\bar{w}$ × 10^2^	$\bar{\sigma_{xx}}$	$\bar{\sigma_{yy}}$	$\bar{\sigma_{xy}}$	$\bar{\sigma_{xz}}$	$\bar{\sigma_{yz}}$
SSSS	Present study (ITSDT)	0°/90°/90°/0°	0.7621	0.5563	0.4170	0.0270	0.2561	0.1223
		0°/60°/60°/0°	0.7091	0.5318	0.2529	0.0384	0.2868	0.1035
		0°/45°/45/°0°	0.6694	0.5195	0.1486	0.0348	0.3073	0.0866
		0°/30°/30°/0°	0.6514	0.5269	0.0761	0.0302	0.3255	0.0658
CCCC	Present study (ITSDT)	0°/90°/90°/0°	0.3724	0.2340	0.2802	0.00138	0.0932	0.1171
		0°/60°/60°/0°	0.3745	0.2411	0.1606	0.00310	0.1203	0.0942
		0°/45°/45/°0°	0.3654	0.2434	0.0892	0.00356	0.1461	0.0732
		0°/30°/30°/0°	0.3460	0.2427	0.0391	0.00316	0.1667	0.0539
SSSC	Present study (ITSDT)	0°/90°/90°/0°	0.6230	0.4542	0.3786	0.0241	0.2108	0.1261
		0°/60°/60°/0°	0.6194	0.4617	0.2452	0.0358	0.2447	0.1053
		0°/45°/45/°0°	0.6139	0.4727	0.1478	0.0337	0.2812	0.0874
		0°/30°/30°/0°	0.6153	0.4949	0.0758	0.0298	0.3111	0.0666
FFCC	Present study (ITSDT)	0°/90°/90°/0°	0.6987	0.1491	0.4889	0.00204	0.0271	0.2017
		0°/60°/60°/0°	0.9761	0.1301	0.4157	0.02197	0.0392	0.1954
		0°/45°/45/°0°	1.3678	0.1145	0.2922	0.02534	0.0513	0.1871
		0°/30°/30°/0°	1.8193	0.1051	0.1509	0.01248	0.0562	0.1769
SSCC	Present study (ITSDT)	0°/90°/90°/0°	0.5108	0.3569	0.3474	0.00655	0.1738	0.1567
		0°/60°/60°/0°	0.5394	0.4003	0.2386	0.00972	0.2222	0.1342
		0°/45°/45/°0°	0.5613	0.4292	0.1472	0.01174	0.2645	0.1096
		0°/30°/30°/0°	0.5802	0.4641	0.0756	0.01247	0.2975	0.0861
FFFC	Present study (ITSDT)	0°/90°/90°/0°	5.8280	0.1572	0.6419	0.00079	0.0254	0.00102
		0°/60°/60°/0°	9.4968	0.1352	0.4419	0.00538	0.0429	0.00908
		0°/45°/45/°0°	14.2504	0.1684	0.2569	0.00235	0.0722	0.00898
		0°/30°/30°/0°	19.1944	0.2036	0.1567	0.00006	0.0524	0.00297

**Table 8:** New results for four-layer laminated porous plate (*p* = 0.1).

Boundary Conditions	Reference (Theory)	Orientation Angle	$\bar{w}$ × 10^2^	$\bar{\sigma_{xx}}$	$\bar{\sigma_{yy}}$	$\bar{\sigma_{xy}}$	$\bar{\sigma_{xz}}$	$\bar{\sigma_{yz}}$
SSSS	Present study (ITSDT)	0°/90°/90°/0°	0.7275	0.5497	0.4023	0.0292	0.2588	0.1199
		0°/60°/60°/0°	0.6766	0.5252	0.2456	0.0408	0.2876	0.1011
		0°/45°/45/°0°	0.6394	0.5135	0.1458	0.0373	0.3074	0.0847
		0°/30°/30°/0°	0.6237	0.5210	0.0753	0.0325	0.3254	0.0642
CCCC	Present study (ITSDT)	0°/90°/90°/0°	0.3504	0.2299	0.2425	0.00151	0.0974	0.1156
		0°/60°/60°/0°	0.3514	0.2370	0.1554	0.00331	0.1248	0.0922
		0°/45°/45/°0°	0.3421	0.2396	0.0867	0.00377	0.1504	0.0719
		0°/30°/30°/0°	0.3234	0.2392	0.0384	0.00335	0.1708	0.0518
SSSC	Present study (ITSDT)	0°/90°/90°/0°	0.5854	0.4492	0.3692	0.0262	0.2131	0.1245
		0°/60°/60°/0°	0.5916	0.4565	0.2399	0.0382	0.2463	0.1029
		0°/45°/45/°0°	0.5869	0.4677	0.1456	0.0362	0.2822	0.0854
		0°/30°/30°/0°	0.5894	0.4897	0.0752	0.0322	0.3117	0.0649
FFCC	Present study (ITSDT)	0°/90°/90°/0°	0.6735	0.1461	0.4873	0.00220	0.0265	0.2024
		0°/60°/60°/0°	0.9443	0.1282	0.4156	0.02291	0.0388	0.1961
		0°/45°/45/°0°	1.3297	0.1133	0.2936	0.02667	0.0512	0.1876
		0°/30°/30°/0°	1.7766	0.1046	0.1529	0.01349	0.0560	0.1780
SSCC	Present study (ITSDT)	0°/90°/90°/0°	0.4883	0.3675	0.3425	0.00720	0.1759	0.1569
		0°/60°/60°/0°	0.5156	0.3961	0.2352	0.01063	0.2239	0.1316
		0°/45°/45/°0°	0.5369	0.4250	0.1454	0.01274	0.2657	0.1086
		0°/30°/30°/0°	0.5558	0.4596	0.0751	0.01346	0.2983	0.0855
FFFC	Present study (ITSDT)	0°/90°/90°/0°	5.7701	0.1537	0.6435	0.00083	0.0247	0.00092
		0°/60°/60°/0°	9.3555	0.1349	0.4484	0.00524	0.0418	0.00885
		0°/45°/45/°0°	14.0553	0.1685	0.2665	0.00231	0.0716	0.00884
		0°/30°/30°/0°	19.0045	0.2025	0.1618	0.00002	0.0533	0.00300

**Table 9:** New results for four-layer laminated porous plate (*p* = 0.2).

Boundary Conditions	Reference (Theory)	Orientation Angle	$\bar{w}$ × 10^2^	$\bar{\sigma_{xx}}$	$\bar{\sigma_{yy}}$	$\bar{\sigma_{xy}}$	$\bar{\sigma_{xz}}$	$\bar{\sigma_{yz}}$
SSSS	Present study (ITSDT)	0°/90°/90°/0°	0.6910	0.5419	0.3861	0.0319	0.2607	0.1180
		0°/60°/60°/0°	0.6428	0.5178	0.2377	0.0439	0.2882	0.1021
		0°/45°/45/°0°	0.6091	0.5067	0.1428	0.0404	0.3073	0.0863
		0°/30°/30°/0°	0.5953	0.5143	0.0743	0.0355	0.3252	0.0665
CCCC	Present study (ITSDT)	0°/90°/90°/0°	0.3276	0.2259	0.2337	0.00167	0.1022	0.1139
		0°/60°/60°/0°	0.3274	0.2329	0.1496	0.00356	0.1297	0.0920
		0°/45°/45/°0°	0.3181	0.2357	0.0839	0.00403	0.1551	0.0716
		0°/30°/30°/0°	0.3003	0.2355	0.0377	0.00358	0.1715	0.0529
SSSC	Present study (ITSDT)	0°/90°/90°/0°	0.5667	0.4436	0.3589	0.0287	0.2154	0.1229
		0°/60°/60°/0°	0.5628	0.4508	0.2341	0.0413	0.2479	0.1039
		0°/45°/45/°0°	0.5930	0.4621	0.1346	0.0392	0.2832	0.0870
		0°/30°/30°/0°	0.5628	0.4838	0.0744	0.0351	0.3121	0.0671
FFCC	Present study (ITSDT)	0°/90°/90°/0°	0.6490	0.1427	0.4855	0.00239	0.0259	0.2031
		0°/60°/60°/0°	0.9127	0.1259	0.4153	0.02393	0.0384	0.1964
		0°/45°/45/°0°	1.2896	0.1122	0.2390	0.02821	0.0509	0.1881
		0°/30°/30°/0°	1.7321	0.1042	0.1552	0.01436	0.0556	0.1782
SSCC	Present study (ITSDT)	0°/90°/90°/0°	0.4653	0.3632	0.3368	0.00799	0.1783	0.1571
		0°/60°/60°/0°	0.4912	0.3915	0.2311	0.01175	0.2257	0.1333
		0°/45°/45/°0°	0.5120	0.4202	0.1434	0.01396	0.2668	0.1109
		0°/30°/30°/0°	0.5309	0.4543	0.0745	0.01475	0.2990	0.0885
FFFC	Present study (ITSDT)	0°/90°/90°/0°	5.7061	0.1502	0.6453	0.00088	0.0240	0.00082
		0°/60°/60°/0°	9.2114	0.1348	0.4558	0.00501	0.0404	0.00799
		0°/45°/45/°0°	13.8409	0.1686	0.2777	0.00222	0.0706	0.00784
		0°/30°/30°/0°	18.7936	0.2012	0.1679	0.00004	0.0538	0.00267

**Table 10:** New results for four-layer laminated porous plate (*p* = 0.3).

Boundary Conditions	Reference (Theory)	Orientation Angle	$\bar{w}$ × 10^2^	$\bar{\sigma_{xx}}$	$\bar{\sigma_{yy}}$	$\bar{\sigma_{xy}}$	$\bar{\sigma_{xz}}$	$\bar{\sigma_{yz}}$
SSSS	Present study (ITSDT)	0°/90°/90°/0°	0.6531	0.5328	0.3687	0.0353	0.2626	0.1164
		0°/60°/60°/0°	0.6081	0.5094	0.2294	0.0471	0.2885	0.1016
		0°/45°/45/°0°	0.5772	0.4991	0.1394	0.0442	0.3070	0.0865
		0°/30°/30°/0°	0.5659	0.5067	0.0732	0.0392	0.3247	0.0672
CCCC	Present study (ITSDT)	0°/90°/90°/0°	0.3041	0.2219	0.2236	0.00186	0.1077	0.1119
		0°/60°/60°/0°	0.3029	0.2288	0.1432	0.00385	0.1353	0.0906
		0°/45°/45/°0°	0.2935	0.2317	0.0705	0.00433	0.1604	0.0707
		0°/30°/30°/0°	0.2768	0.2317	0.0368	0.00386	0.1798	0.0524
SSSC	Present study (ITSDT)	0°/90°/90°/0°	0.5370	0.4373	0.3475	0.0319	0.2178	0.1215
		0°/60°/60°/0°	0.5331	0.4443	0.2477	0.0451	0.2495	0.1035
		0°/45°/45/°0°	0.5306	0.4556	0.1402	0.0429	0.2841	0.0871
		0°/30°/30°/0°	0.5351	0.4769	0.0734	0.0387	0.3123	0.0678
FFCC	Present study (ITSDT)	0°/90°/90°/0°	0.6237	0.1392	0.4834	0.02643	0.0253	0.2039
		0°/60°/60°/0°	0.8953	0.1238	0.4149	0.02512	0.0378	0.1969
		0°/45°/45/°0°	1.2480	0.1112	0.2969	0.02986	0.0504	0.1887
		0°/30°/30°/0°	1.6855	0.1038	0.1582	0.01562	0.0550	0.1787
SSCC	Present study (ITSDT)	0°/90°/90°/0°	0.4416	0.3583	0.3301	0.00899	0.1809	0.1573
		0°/60°/60°/0°	0.4659	0.3863	0.2265	0.01314	0.2275	0.1338
		0°/45°/45/°0°	0.4863	0.4147	0.1411	0.01514	0.2679	0.1118
		0°/30°/30°/0°	0.5051	0.4481	0.0737	0.01631	0.2995	0.0895
FFFC	Present study (ITSDT)	0°/90°/90°/0°	5.6497	0.1463	0.6472	0.00943	0.0232	0.00071
		0°/60°/60°/0°	9.0503	0.1347	0.4647	0.00470	0.0386	0.00731
		0°/45°/45/°0°	13.5975	0.1685	0.2909	0.00208	0.0691	0.00714
		0°/30°/30°/0°	18.5514	0.1993	0.1754	0.00013	0.0542	0.00241

**Table 11:** Validation of two-layer laminated plate.

a/h	Reference	Theory	Normalized Deflection		
			SSSS	SSCC	SSFF
10	Present study (16 × 16)	ITSDT	1.154	0.534	1.866
	Liu et al. [4]	FSDT	1.245	0.665	2.031
	Khdeir and Reddy [48]	FSDT	1.237	0.656	2.028
	Vel and Batra [49]	Analytical load	1.227	0.648	2.026

**Table 12:** New results for two-layer laminated plate with various boundary conditions.

Boundary Conditions	Reference	Theory	Orientation Angle	Normalized Deflections			
				e = 0	e = 0.1	e = 0.2	e = 0.3
SSSS	Present study	ITSDT	0°/90°	1.154	1.129	1.095	1.058
			0°/60°	0.974	0.948	0.920	0.887
			0°/45°	0.868	0.841	0.813	0.780
			0°/30°	0.765	0.738	0.709	0.678
SSCC	Present study	ITSDT	0°/90°	0.534	0.516	0.506	0.491
			0°/60°	0.564	0.549	0.534	0.518
			0°/45°	0.601	0.585	0.567	0.548
			0°/30°	0.636	0.615	0.593	0.569
SSFF	Present study	ITSDT	0°/90°	1.866	1.854	1.842	1.829
			0°/60°	1.711	1.682	1.648	1.610
			0°/45°	1.463	1.421	1.375	1.323
			0°/30°	1.132	1.087	1.036	0.985
SSSC	Present study	ITSDT	0°/90°	0.770	0.751	0.732	0.709
			0°/60°	0.731	0.712	0.692	0.670
			0°/45°	0.719	0.698	0.676	0.652
			0°/30°	0.699	0.675	0.650	0.622
SSFS	Present study	ITSDT	0°/90°	1.560	1.536	1.504	1.478
			0°/60°	1.340	1.305	1.279	1.241
			0°/45°	1.194	1.158	1.119	1.075
			0°/30°	1.023	0.983	0.940	0.892
SSFC	Present study	ITSDT	0°/90°	1.085	1.068	1.050	1.029
			0°/60°	0.998	0.978	0.895	0.931
			0°/45°	0.983	0.956	0.925	0.892
			0°/30°	0.916	0.882	0.845	0.804

**Table 13:** Validation of three-layer square laminated plate.

a/h	Reference	Theory	Normalized Deflection		
			SSSS	SSCC	SSFF
5	Present study (16 × 16)	ITSDT	1.596	1.018	5.197
	Liu et al. [4]	FSDT	1.562	1.201	5.249
	Vel and Batra [49]	Analytical load	1.525	1.180	5.307
10	Present study (16 × 16)	ITSDT	0.782	0.441	4.424
	Liu et al. [3]	FSDT	0.753	0.448	4.335
	Vel and Batra [49]	Analytical load	0.753	0.446	4.453

**Table 14:** New results for two-layer laminated plate with various boundary conditions (a/h = 5).

Boundary Conditions	Reference	Theory	Orientation Angle	Normalized Deflections			
				e = 0	e = 0.1	e = 0.2	e = 0.3
SSSS	Present study	ITSDT	0°/90°/0°	1.596	1.492	1.383	1.270
			0°/60°/0°	1.412	1.323	1.232	1.139
			0°/45°/0°	1.288	1.209	1.130	1.050
			0°/30°/0°	1.268	1.188	1.109	1.026
SSCC	Present study	ITSDT	0°/90°/0°	1.018	0.958	0.889	0.823
			0°/60°/0°	1.081	1.015	0.945	0.871
			0°/45°/0°	1.147	1.075	0.999	0.921
			0°/30°/0°	1.192	1.116	1.036	0.955
SSFF	Present study	ITSDT	0°/90°/0°	5.197	5.098	4.995	4.893
			0°/60°/0°	4.297	4.109	3.910	3.701
			0°/45°/0°	2.792	2.628	2.458	2.281
			0°/30°/0°	1.737	1.627	1.514	1.399
SSSC	Present study	ITSDT	0°/90°/0°	1.279	1.199	1.114	1.080
			0°/60°/0°	1.239	1.162	1.082	0.998
			0°/45°/0°	1.216	1.141	1.051	0.984
			0°/30°/0°	1.230	1.152	1.072	0.990
SSFS	Present study	ITSDT	0°/90°/0°	3.544	3.396	3.238	3.066
			0°/60°/0°	2.362	2.243	2.118	1.988
			0°/45°/0°	1.875	1.767	1.656	1.541
			0°/30°/0°	1.493	1.402	1.306	1.208
SSFC	Present study	ITSDT	0°/90°/0°	2.250	2.143	2.028	1.905
			0°/60°/0°	1.973	1.869	1.762	0.646
			0°/45°/0°	1.769	1.664	1.554	1.440
			0°/30°/0°	1.453	1.361	1.265	1.167

**Table 15:** New results for two-layer laminated plate with various boundary conditions (a/h = 10).

Boundary Conditions	Reference	Theory	Orientation Angle	Normalized Deflections			
				e = 0	e = 0.1	e = 0.2	e = 0.3
SSSS	Present study	ITSDT	0°/90°/0°	0.782	0.744	0.705	0.665
			0°/60°/0°	0.728	0.699	0.668	0.637
			0°/45°/0°	0.681	0.656	0.631	0.605
			0°/30°/0°	0.677	0.652	0.627	0.601
SSCC	Present study	ITSDT	0°/90°/0°	0.441	0.412	0.381	0.350
			0°/60°/0°	0.483	0.455	0.426	0.395
			0°/45°/0°	0.541	0.513	0.485	0.456
			0°/30°/0°	0.603	0.577	0.550	0.522
SSFF	Present study	ITSDT	0°/90°/0°	4.424	4.379	4.370	4.340
			0°/60°/0°	3.408	3.301	3.179	3.044
			0°/45°/0°	1.848	1.774	1.694	1.608
			0°/30°/0°	0.963	0.927	0.889	0.851
SSSC	Present study	ITSDT	0°/90°/0°	0.578	0.543	0.507	0.470
			0°/60°/0°	0.589	0.559	0.528	0.495
			0°/45°/0°	0.605	0.582	0.552	0.524
			0°/30°/0°	0.635	0.613	0.587	0.560
SSFS	Present study	ITSDT	0°/90°/0°	2.782	2.699	2.605	2.499
			0°/60°/0°	1.519	1.472	1.424	1.307
			0°/45°/0°	1.119	1.078	1.036	0.992
			0°/30°/0°	0.817	0.786	0.755	0.722
SSFC	Present study	ITSDT	0°/90°/0°	1.312	1.257	1.200	1.134
			0°/60°/0°	1.112	1.065	1.016	0.965
			0°/45°/0°	0.988	0.944	0.899	0.851
			0°/30°/0°	0.775	0.743	0.711	0.677

## Data Availability

Data are contained within the article.

## Abbreviations

ITSDT	Improved third-order shear deformation theory
FSDT	First-order shear deformation theory
HSDT	Higher-order shear deformation theory
HZT	Higher-order zigzag theory
